# Quality of Life in Adult Patients Receiving Cervical Fusion for Fresh Subaxial Cervical Injury: The Role of Associated Spinal Cord Injury

**DOI:** 10.1155/2021/9931535

**Published:** 2021-05-15

**Authors:** Panagiotis Korovessis, Evangelia Mpountogianni, Vasileios Syrimpeis, Maria Andriopoulou, Alkis Korovesis

**Affiliations:** ^1^Orthopedic Department, General Hospital “Agios Andreas”, Patras, Greece; ^2^Open University, Patras, Greece

## Abstract

**Purpose:**

To study postoperative Health-Related Quality of Life (HRQOL) after instrumented fusion for fresh subaxial cervical trauma and the effect of spinal cord injury (SCI).

**Methods:**

From a total of 65 patients, 17 (26%) patients suffered on admission from SCI. Twenty-five patients underwent anterior, 25 posterior, and 15 circumferential cervical surgery for a single cervical injury. Sagittal roentgenographic parameters were measured in 65 age-matched asymptomatic controls and in patients on admission, eight months postoperatively and at final follow-up (lower C_2_-C_7_ curvature, cervical sagittal vertical axis (cSVA), spinocranial angle (SCA), T_1_-slope, neck tilt (NT), thorax inlet angle (TIA), cervical tilt (CT), cranial tilt (CrT), and occiput–C_2_ angle (C_0_-C_2_)). In the last evaluation, SCI patients were compared with their counterparts without SCI using national validated HRQOL instruments (SF-36 and neck disability index (NDI)).

**Results:**

Fusion included an average of 3 vertebrae (range 2-4 vertebrae). All 65 patients were followed for an average of 5.5 years, (range 3-7 years) postoperatively. In the last evaluation, 10 (15.4%) patients with incomplete SCI improved postoperatively at 1-2 grades. At the last observation, patients with SCI showed poorer HRQOL scores than their counterparts without SCI. In particular, each SF-36 domain score was correlated with SCA, T_1_-slope, cSVA, and CT. At baseline, patients showed higher NT, CrT, and C0-C2 angle than controls. Eight months postoperatively, cSVA, NT, TIA, and cranial tilt (CrT) were increased in patients. In the last observation, there was difference in the sagittal roentgenographic parameters between patients with SCI compared to those without SCI. Patients aged ≥55 years had postoperatively increased cSVA, NT, and CrT compared to their younger counterparts.

**Conclusion:**

At the final observation, HRQOL scores were lower in patients with SCI than in their non-SCI counterparts, obviously because of the associated neurologic impairment. SF-36 scores correlated with several sagittal roentgenographic parameters. These correlations should be taken in consideration by spine surgeons when performing cervical spine surgery for fresh cervical spine injuries.

## 1. Introduction

Relationships between sagittal lumbopelvic alignment and HRQOL measures have been shown in spinal deformity surgery [1]. Sagittal alignment was studied after degenerative cervical spine disease surgery [2–12], but to our knowledge, less attention was paid to the difference in postoperative HRQOL between SCI and non-SCI patients who underwent cervical spine fixation after fresh subaxial injury of surgery.

The purpose of this investigation was to correlate postoperative HRQOL and presence of SCI and postoperative sagittal cervical balance with HRQOL (NDI and SF-36).

## 2. Patients and Methods

**Figure 1:**
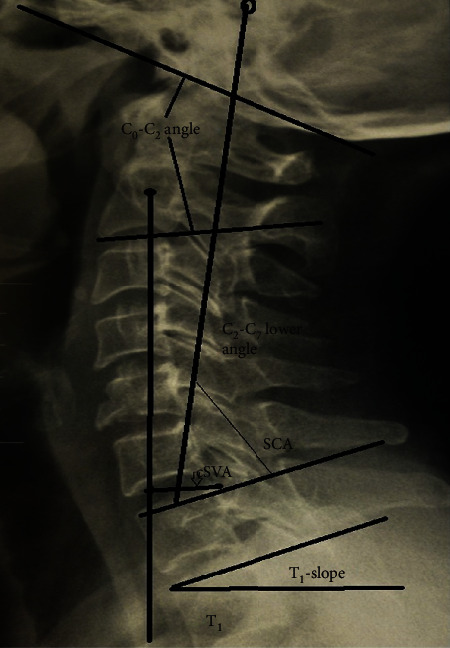
Sagittal roentgenographic parameters on standing lateral skull and cervical roentgenogram. C_0_-C_2_ = higher cervical curvature; C_2_-C_7_ = lower cervical curvature; cSVA = sagittal vertebral axis; T_1_-slope; SCA: spinocranial angle.

**Figure 2:**
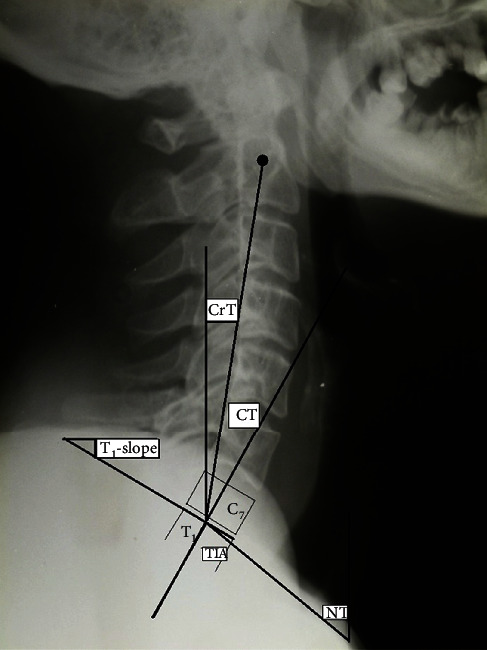
Sagittal roentgenographic parameters on standing lateral skull and cervical roentgenogram. CrT = cranial tilt; CT = cervical tilt; NT = neck tilt; TIA = thorax inlet angle.

In the period between January 2012 and December 2015, a total of 71 consecutive adult patients were admitted to the authors' department with fresh unstable subaxial cervical injuries. The power analysis with power 80% showed a total required number of individuals for two independent groups (patients and asymptomatic individuals) was 65. Sixty-five consecutive patients underwent primary cervical spine fixation by an experienced senior orthopedic spine surgeon in a single institution within the first 48 hours following trauma with the exception of cases of emergency life-threatening conditions in other organs that needed surgery. The inclusion criteria were as follows: unstable single subaxial cervical injury and age ≥ 18 years, while the exclusion criteria were as follows: injuries required occipitocervical and cervicothoracic fusion, nontraumatic instability, previous cervical spinal surgeries, history of degenerative cervical myelopathy, and serious psychiatric disease. If the landmarks (skull, cervicothoracic junction) necessary for distinct roentgenographic measurements were poorly discernable, these patients were excluded from the study. Four (5.6%) patients died in the first 3 months following trauma for different reasons related either to associated injuries or comorbidities and were excluded for the final evaluation. Two (2.8%) additional patients were excluded from the statistical analysis because of poorly discernable cervicothoracic junction. We conducted a retrospective radiographic and HRQOL analysis of the remainder 65 consecutive adult patients, 48 men and 17 women, who underwent anterior, posterior, or 360° surgery. The causes of cervical trauma were fall in 14 (21.5%) and traffic accident in 51 (78.5%) patients. Seventeen patients (26%) suffered on admission from SCI (ASIA A-C). There were associated injuries in 19 (28.6%) patients (pelvic, extremities, abdomen blunt, and cerebral trauma). The most common cervical injury was the AO/type-C [3] in 16 (24.6%) patients (Table 1). Standing digital cervical spine roentgenograms from 100 asymptomatic individuals, aged 52 ± 17 years derived from the database of this institution without a history of spinal injury, tumor, infection, ankylosing spondylitis, and previous spinal surgery, were subsequently selected as controls to match to patients' age. According to the roentgenographic history charts, the indications for cervical spine roentgenogram in the controls were migraine, headache, and vertigo. All spinal roentgenograms in this institution in ambulatory individuals are made in standing position with the upper extremities flexed to the shoulder in relaxed position with gaze straight forward adjust for their height. All patients with SCI were examined radiologically in sitting position postoperatively. At the final observation, the HRQOL scores of the patients with SCI were compared with those of the neurologically intact patients using validated and nationally adapted questionnaires (neck disability index (NDI) [4] and SF-36 [5]). The radiographs were taken on a digital X-ray system (eFilm Workstation 4.2 software, Merge Healthcare, Hartland, WI USA) and analyzed using commercial software that allows for measurements with 0.1 mm increments and enhancing of vertebral levels at the cervicothoracic junction. The lateral cervical roentgenographic parameters were measured in standing position in the controls and in supine position in patients on admission; 8-10 months postoperatively in standing position for ambulatory patients and sitting for SCI patients position and at the final evaluation in standing (non-SCI patients) or sitting position (SCI patients) were as follows: (1) lower C_2_-C_7_ curvature (angle formed from the lower plates of C_2_ and C_7_ vertebrae); (2) spinocranial angle (SCA): the angle is defined between the C_7_-slope and the straight line joining the middle of the upper C_7_-endplate and the middle of the sella turcica; (3) T_1_-slope: angle formed between a horizontal line and the superior endplate of T_1_-vertebra; (4) cSVA: the distance from the posterior/superior corner of C_7_ vertebral body to the plumbline from the C_2_ centroid; (5) neck tilt (NT): angle formed by the reference vertical line drawn in the upper end of the sternum and a line connecting the center of the T_1_-upper endplate and the upper end of the sternum; (6) thorax inlet angle (TIA): angle formed by a line perpendicular to the superior endplate of T_1_ and a line connecting the T_1_-upper endplate and the upper end of the sternum; (7) cervical tilt (CT): angle between two lines, both originating from the center of the T_1_-upper endplate; one is vertical to the T_1_-upper endplate; one is vertical to the T_1_-upper endplate, and the other passes through the tip of the dens; (8) cranial tilt (CrT): the angle between two lines, both originating from the center of the T_1_ upper endplate, with one passing through the dens and the other being a vertical line; and (9) C_0_-C_2_ (occiput–C2 angle): angle created by McGregor's line and the inferior surface of the axis (Figures 1 and 2). In the last observation, personal interview and physical examination were made in all patients. To test the inter- and intraobserver agreement, the sagittal cervical parameters were measured blinded in 30 randomly selected digital lateral radiographs from the controls and from 20 patients twice within a two-week interval by two independent orthopedic surgeons, who did not participate in the surgeries, using the Surgimap software (Surgimap Spine, Nemaris Inc., New York, USA). Additional radiological evaluation (roentgenograms, CT scans) of the cervical spine was made 8-10 months postoperatively to evaluate spinal fusion. All 65 patients were physically examined by the 4^th^ author and completed the NDI and SF-36 questionnaires.

The ethics committee of this institution approved the study protocol, and informed consent was obtained from the study participant or the person authorized to give consent. The Statistical Package for the Social Sciences (IBM SPSS Statistics version 25, SPSS Inc., Chicago, Illinois, United States) software was used. All data for the patients and controls in this study are in the central PACS documentation archives of the Orthopedic Department, General Hospital Patras Greece.

### 2.1. Surgical Techniques

The patient was positioned in prone position for a posterior approach with head on a headrest without skull traction or in a supine position for an anterior approach. Closed manual or reduction was applied in cases with dislocations or gross sagittal displacement under image intensifier and continuous neuromonitoring. Preoperatively cervical alignment and immobilization were maintained with a cervical orthosis. The lateral cervical alignment was controlled with a biplane image intensifier and measured intraoperatively digitally. Facet/pedicle screw fixation plus appropriately contoured rods were used for stabilization in the posterior approach. Discectomy, vertebrectomy, and a cage filled with bone graft plus lordotic contoured plate were used in the anterior surgery. A soft collar was used in all patients immediately postoperatively and a Minerva brace for mobilization of the patients for 6-8 weeks.

### 2.2. Statistical Data Analysis

The reproducibility and repeatability of all roentgenographic measurements were evaluated using the kappa value. The skewness and kurtosis tests were used to test the data frequency in both groups. The paired *t*-test was used for comparison of the same continuous variable and unpaired *t*-test for different continuous variable change between two periods of observation. One-way ANOVA was principally used to compare the difference of means of continuous variables between 2 or more subgroups of each categorical variable. The bivariate Pearson correlation coefficient (*r*) was used to correlate pairs of continuous variables.

## 3. Results

Interobserver and intraobserver *k*-values for sagittal roentgenographic parameters ranged between 0.99 and 1.0. There were no kurtosis or skewed longitudinal data.

Twenty-five (38.5%) patients underwent posterior surgery, 25 (38.5%) anterior, and the remainder 15 (23%) 360° surgery.

Three (4.6%) patients were reoperated within the first month following primary surgery for deep spinal infection.

All spines showed completed fusion 8-10 months postoperatively.

All 65 patients were available for the final evaluation in an average of 5.5, range 3-7 years postoperatively.

In the last evaluation, 7 (10.8%) patients showed residual neurologic deficit (ASIA A-C), while 10 (15.4%) patients with preoperative incomplete SIC improved at 1-2 ASIA grades postoperatively.

### 3.1. HRQOL (Patients)

The NDI averaged 23.8% ± 25% (range 0-72) and was not related to any sagittal parameter (Table 2).

SF-36 scores significantly correlated with SCA, T_1_-slope, cSVA, and CT (Table 2).

SCI patients showed higher NDI and lower SF-36 scores than neurologically intact patients at the final observation (Table 3).

Significant correlations were shown in the controls between pairs of sagittal roentgenographic parameters (Table 4).

On admission, patients showed higher NT, CrT, and C_0_-C_2_ than controls (Table 5).

Eight months postoperatively, cSVA, NT, TIA, and CrT were more in patients (Table 5).

Lower C2-C7 curvature (*p* = 0.001), T_1_-slope (*p* = 0.001), TIA (*p* = 0.008), and CrT (*p* = 0.006) increased postoperatively (Table 6).

Higher C_0_-C_2_ curvature (*p* = 0.029) increased between the follow-up of 8-10 months postoperatively to the final observation (Table 7).

At the final postoperative observation, T_1_-slope was correlated with lower C_2_-C_7_ cervical curvature (*p* = 0.015), cSVA (*p* = 0.012), TIA (*p* = 0.002), CT (*p* < 0.0001), and CrT (*p* < 0.0001). (Table 7). Older (≥55 years) patients showed cSVA < 4 cm but increased NT (*p* = 0.006) and CrT (*p* = 0.038) compared to their younger counterparts (≤54 years).

In the last observation, the measured sagittal roentgenographic parameters did not differ between patients with SCI and those without SCI (one-way ANOVA).

## 4. Discussion

Although sagittal alignment parameters associated with reconstructive surgery in cervical degenerative disease are available, to our knowledge, no postoperative sagittal alignment parameters are defined in patients with fresh subaxial cervical injuries who underwent surgical reduction and stabilization, while no data exist regarding correlation SCI and HRQOL [9].

Although some authors postulated that loss of cervical lordosis is associated with pain and disability, no correlation was found at the final observation in the patients of our series between lower C_2_-C_7_ cervical lordosis and NDI and SF-36 scores [1, 10]. In our series, 5% of the controls and 5% of the patients showed postoperatively kyphotic cervical spine.

Although several papers reported correlations between HRQOL scores and sagittal cervical spine alignment [11–13], there is no such data regarding postoperative sagittal alignment in subaxial cervical injuries. Koller et al. [9] in a series of 28 adult patients who received surgery for unstable subaxial injuries without neurological deficit reported 5.5 years postoperatively a NDI of 12.4 ± 12.7%, this being less than our NDI score of 23.8 ± 25%, with similar follow-up. This was obviously due to the inclusion of patients with SCI in our series. Although it was anticipated, in our series, patients with SCI showed higher disability NDI and lower SF-36 scores than their neurologically intact counterparts. In Koller et al.'s study, the SF-36 mean physical and mental component summary scores were better than in our patients, obviously because of the inclusion of patients with SCI in our series [9]. Postoperative SF-36 scores were higher in our patients with balanced cervical spine defined as low SCA, T_1_-slope, cSVA, and CT. In our series, in the final observation, disability (NDI) was much higher than the reported average of 6.98% in the general population [14]. Koller et al. [9] showed a postoperative cervical lordosis of −24.3 ± 13.3° that was close to our lower C_2_-C_7_ lordosis (−21.87 ± 9.5°).

LeHuec et al. were the first who defined the CT and SCA in cervical balance [15]. They showed that economic sagittal balance in asymptomatic population was defined by a SCA angle of 83° ± 9° [15], higher than that in both our patients (74 ± 9°) and controls (73 ± 10°). This may be due to different populations (different ethnicity, samples, etc.). In our study, we also used CT and SCA in defining sagittal balance but we selected the T_1_-slope instead of the C_7_-slope used by LeHuec et al. for sagittal cervical spine alignment [15]. In our controls and patients, T_1_-slope was negatively correlated with lower C_2_-C_7_ curvature. We speculate that cervical spine keeps a lordotic lower C_2_-C_7_ curvature adapting it to the T_1_-slope.

LeHuec et al. introduced CrT as the postural variable providing information about the spatial position of the head [15]. In our series, postoperatively CrT was increased compared to the controls, indicating no change in head spatial position following surgical stabilization of subaxial cervical spine.

We speculate that the increased cSVA, NT, and CrT in older patients in our study should be compensatory mechanisms of the cervical spine occurring with ageing.

The upper C_0_-C_2_ angle increased significantly (*p* = 0.029) at the final postoperative observation. The latter allows us to speculate that this is an ongoing compensatory response of the cervical spine to keep our patients the horizontal gaze. Adaptation changes at the upper C_0_-C_2_ and lower C_2_-C_7_ angles are the final “physiological” compensatory mechanism against thoracolumbar deformity of the patients and volunteers to keep horizontal gaze [16]. In our patients, the C_0_-C_2_ angle postoperatively averaged −19 ± 9°, identical with that in our controls but higher than that reported in asymptomatic individuals in other studies (14-16°) [9, 17].

Maintaining lordotic the lower C_2_-C_7_ curvature improves the NDI and SF-36 scores up to 2 years postoperatively after anterior fusion for degenerated cervical spine [17]. In our series, early reduction and fusion maintained lordotic or even improved the lower C_2_-C_7_ curvature, close to asymptomatic individuals. Some authors [9, 18] reported that cSVA and T_1_-slope had an impact on the NDI scores following mono- and multisegmental anterior cervical fusion for degenerative disease. Similarly, the NDI score was negatively correlated with T_1_-slope and cSVA.

There is a discrepancy among different reports regarding the influence of improved cervical lordosis after anterior cervical fusion and arthroplasty and HRQOL improvement [19, 20]. However, the findings of our study showed no correlation between lower C_2_-C_7_ lordosis and HRQOL scores.

There is some controversy in the literature concerning spinopelvic alterations in SCI patients with upper thoracic injury level [21, 22], while there is a paucity of literature that directly addresses sagittal imbalance in paraplegic patients. Some studies have shown significant alterations of the sagittal thoracolumbopelvic but not of the cervical alignment parameters in the nonambulatory paraplegic patients compared to ambulatory patients, depending on the level of the SCI [21–23].

There are several limitations in our study. (1) We analyzed a relatively small cohort of 65 patients. We are satisfied that we were able to identify sound statistical differences between patients and asymptomatic controls and to correlate sagittal roentgenographic parameters and HRQOL scores. (2) The smaller number of female patients was due to the fact that women rarely are involved in such injuries; (3) limitation would be that this database is surgeon maintained; however, the measurement of the digital roentgenograms was made by unbiased observes, while the validity was tested appropriately and found excellent. (4) The lack of sagittal lumbosacral roentgenographic measurements particularly in the patients with SCI was technically not possible in our facilities. (5) No sitting whole spine roentgenograms from controls and patients were taken as it would be not possible and ethical in controls.

In conclusion, NDI and SF-36 scores were lower in SCI than in their non-SCI patients, obviously because of associated neurologic impairment. SF-36 scores correlated with several sagittal roentgenographic parameters. Surgeons should take into consideration these correlations when performing cervical spine stabilization for fresh spinal injury.

## Figures and Tables

**Table 1:** Cervical injury classification in 65 patients with fresh cervical trauma.

Type of injury	*N* (patients)	%
AO type A4	3	4.62
AO type B1	4	6.15
AO type B2	2	13.85
AO type B3	10	15.38
AO type B4	9	13.85
AO type C	16	24.62
AO type F4	10	15.38
Combined cervical injury	4	6.15

Total	65	100

**Table 2:** Pearson correlation matrix between sagittal balance and HRQOL scores in 65 patients in the last evaluation.

Parameters	Lower C_2_-C_7_ curvature	SCA	T1-slope	cSVA	NT	TIA	CT	CrT	C_0_-C_2_^+^
NDI score	*r* = 0.059	*r* = 0.090	*r* = 0.292	*r* = 0.375	*r* = 0.142	*r* = 0.289	*r* = 0.274	*r* = 0.170	*r* = 0.090
Physical functioning (PH)^∗∗^	*r* = 0.0105	*r* = 0.269	*r* = 0.094	*r* = 0.217	*r* = 0.147	*r* = 0.164	*r* = 0.155	*r* = 0.026	*r* = 0.261
Role limitation PH^∗∗^	*r* = 0.052	*r* = 0.251	*r* = 0.482, *p* = 0.017	*r* = 0.346	*r* = 0.054	*r* = 0.275	*r* = 0.493, *p* = 0.014	*r* = 0.232	*r* = 0.105
Limitations due to role^∗∗^ emotional problems	*r* = 0.043	*r* = 0.203	*r* = 0.406, *p* = 0.049	*r* = 0.381	*r* = 0.026	*r* = 0.245	*r* = 0.472, *p* = 0.020	*r* = 0.125	*r* = 0.011
Energy/fatigue^∗∗^	*r* = 0.199	*r* = 0.148	*r* = 0.275	*r* = 0.468, *p* = 0.021	*r* = 0.130	*r* = 0.269	*r* = 0.199	*r* = 0.234	*r* = 0.020
Emotional well-being^∗∗^	*r* = 0.144	*r* = 0.011	*r* = 0.359	*r* = 0.551, *p* = 0.005	*r* = 0.040	*r* = 0.205	*r* = 0.288	*r* = 0.270	*r* = 0.015
Social functioning ^∗∗^	*r* = 0.024	*r* = 0.011	*r* = 0.379	*r* = 0.392	*r* = 0.155	*r* = 0.355	*r* = 0.332	*r* = 0.250	*r* = 0.108
Pain^∗∗^	*r* = 0.053	*r* = 0.103	*r* = 0.462, *p* = 0.023	*r* = 0.510, *p* = 0.011	*r* = 0.093	*r* = 0.256	*r* = 0.414, *p* = 0.044	*r* = 0.293	*r* = 0.045
General health (GH)^∗∗^	*r* = 0.100	*r* = 0.161	*r* = 0.211	*r* = 0.365	*r* = 0.170	*r* = 0.256	*r* = 0.119	*r* = 0.220	*r* = 0.025
GH change^∗∗^	*r* = 0.125	*r* = 0.480, *p* = 0.018	*r* = 0.388	*r* = 0.155	*r* = 0.243	*r* = 0.081	*r* = 0.392	*r* = 0.191	*r* = 0.161

+ = occiput-C_2_ angle; ∗∗ = SF-36 domain; CT = cervical tilt; CrT = cranial tilt. *p* value is added in significant *r* values only.

**Table 3:** NDI and SF-36 scores in neurologically intact and spinal cord injury patients in the final observation.

Patients	NDI	Physical functioning	Role limitations due to physical health	Role limitations due to emotional problems	Energy/fatigue	Emotional well-being	Social functioning	Pain	General health	General health change
Neurologically intact	13.22 ± 16	83.88 ± 25.9	93.05 ± 20.65	100.0	100.0	74.72 ± 10	85.83 ± 22.59	95.83 ± 22.59	63.88 ± 21.4	61.11 ± 19.59
Spinal cord injury (SCI)	47.5 ± 26	31.87 ± 32.9	46.87 ± 50.77	50 ± 53.45	34.37 ± 15.68	48 ± 15.26	33.33 ± 14.43	50 ± 43.58	25 ± 13	43.75 ± 16.67
^∗^ *p* value	0.007	0.0022	0.0377	0.0331	0.00024	0.0011	0.00551	0.208	0.00001	0.04124

^∗^Unpaired *t*-test.

**Table 4:** Pearson correlation matrix between pairs of sagittal roentgenographic parameters in 100 controls.

	Lower C_2_-C_7_ curvature	SCA	T_1_-slope	cSVA	NT	TIA	CT	CrT	C_0_-C_2_ angle
Age	*r* = −0.476, *p* = 0.011	*r* = −0.196	*r* = 0.462, *p* = 0.013	*r* = 0.307	*r* = 0.073	*r* = 0.451, *p* = 0.016	*r* = 0.356	*r* = 0.334	*r* = 0.031
Lower C_2_-C_7_ curvature	1	*r* = −0.159	*r* = −0.524, *p* = 0.004	*r* = 0.123	*r* = 0.133	*r* = −0.362	*r* = −0.706, *p* < 0.0001	*r* = 0.025	*r* = 0.516, *p* = 0.005
SCA		1	*r* = −0.321	*r* = −0.643, *p* < 0.0001	*r* = −0.232	*r* = −439, *p* = 0.019	*r* = 0.065	*r* = −0.650, *p* < 0.0001	*r* = −0.352
T_1_-slope			1	*r* = 0.475, *p* = 0.012	*r* = −0.194	*r* = 0.733, *p* < 0.0001	*r* = 0.823, *p* < 0.01	*r* = 0.651, *p* < 0.0001	*r* = 0.603
cSVA				1	*r* = 0.278	*r* = 0.628, *p* < 0.0001	*r* = −0.87	*r* = 0.920, *p* < 0.0001	*r* = 0.163
NT					1	*r* = 0.525, *p* = 0.004	*r* = −0.385, *p* = 0.043	*r* = 0.175	*r* = 0.221
TIA						1	*r* = 0.448, *p* = 0.017	*r* = 0.686, *p* < 0.0001	*r* − = 0.064
CT							1	*r* = 0.105	*r* = −0.273
CrT								1	*r* = 0.185

**Table 5:** Comparative roentgenographic parameter values in 65 patients pre- and postop vs. versus 100 controls.

	Lower C_2_-C_7_ curvature	SCA	T_1_-slope	cSVA	NT	TIA	CT	CrT	C_0_-C_2_
Patients preop	−18.79 ± 12.69	73.72 ± 11.32	25.15 ± 10.6	2.97 ± 2.42	55.62 ± 10.97	80.77 ± 12.41	14.95 ± 9.6	10.19 ± 7.94	19.99 ± 7.95
Controls	−19.9 ± 13	74.72 ± 9	28.6 ± 9.8	1.5 ± 0.83	48.1 ± 9.3	77.1 ± 13	18.9 ± 8	9.8 ± 6.5	19 ± 9
*p* value pts vs. controls	0.0134	0.5640	0.0000	0.1067	0.0032	0.8502	0.4827	0.0044	0.0000
Patients postop^∗^	−21.87 ± 9.5	73.01 ± 10.14	31.55 ± 12.3	3.3 ± 1.86	55.56 ± 11.39	86.11 ± 16.85	17.22 ± 9.46	14.32 ± 7.85	19.48 ± 8.70
*p* value pts vs. controls	0.0765	0.256	0.2168	0.0000	0.0006	0.0017	0.2549	0.0013	0.6326

**Table 6:** Changes of sagittal roentgenographic parameters preoperatively to follow-up in 65 patients.

	Curvature lower C_2_-C_7_	SCA	T_1_-slope	cSVA (cm)	NT	TIA	CT	CrT	C_0_-C_2_
Preop	−18.79 ± 12.69	73.72 ± 11.32	25.15 ± 10.6	2.97 ± 2.42	55.62 ± 10.97	80.77 ± 12.41	14.95 ± 9.6	10.19 ± 7.94	19.99 ± 7.95
Postop	−21.87 ± 9.5	73.01 ± 10.14	31.55 ± 12.3	3.3 ± 1.86	55.56 ± 11.39	86.11 ± 16.85	17.22 ± 9.46	14.32 ± 7.85*o*	19.48 ± 8.70
*p* value pre/post	0.001	0.852	0.001	0.4	0.452	0.008	0.160	0.006	0.671
F/up	−16.49 ± 12.29	67.61 ± 17.92	29.85 ± 11.36	3.18 ± 2.10	53.28 ± 10.94	83.13 ± 14.91	18.44 ± 6.83	11.41 ± 6.91	23.72 ± 8.81
*p* value post/fup	0.820	0.415	0.716	0.239	0.608	0.519	0.223	0.449	0.029
*p* value preop/fup	0.91	0.142	0.04	0.859	0.37	0.85	0.101	0.01	0.523

**Table 7:** Pearson correlation matrix between pairs of sagittal roentgenographic parameters in 65 patients at the final observation.

	SCA	T_1_-slope	cSVA	NT	TIA	CT	CrT	C_0_-C_2_ angle
Lower C_2_-C_7_ curvature	*r* = 0.155	*r* = −0.597, *p* = 0.015	*r* = −0.046	*r* = 0.098	*r* = −0.378	*r* = −0.671, *p* = 0.004	*r* = −0.340	*r* = 0.308
SCA	1	*r* = −0.073	*r* = 0.150	*r* = −0.280	*r* = −0.261	*r* = −0.195	*r* = 0.074	*r* = 0.241
T_1_-slope		1	*r* = 0.577, *p* = 0.012	*r* = −0.107	*r* = 0.683, *p* = 0.002	*r* = 0.823, *p* < 0.0001	*r* = 0.828, *p* < 0.0001	*r* = 0.282
cSVA			1	*r* = −0.031	*r* = 0.416	*r* = 0.414	*r* = 0.537, *p* = −0.022	*r* = 0.594, *p* = 0.009
NT				1	*r* = 0.653, *p* = 0.003	*r* = 0.093	*r* = −267	*r* = 0.350
TIA					1	*r* = 0.696, *p* = 0.001	*r* = 0.435	*r* = 0.472, *p* = 0.048
CT						1	*r* = 0.364	*r* = 0.082
CrT							1	*r* = 0.382

## Data Availability

All data for the patients and controls in this study are in the central PACS documentation archives of the Orthopedic Department, General Hospital Patras Greece.
